# Supplementary material for: Enhanced Skin Permeation of 5-Fluorouracil through Drug-in-Adhesive Topical Patches
Source: Pharmaceutics. 2024 Mar 10;16(3):379. doi: 10.3390/pharmaceutics16030379 (PMC10975131; doi:10.3390/pharmaceutics16030379)
Supplement: Supplementary file 1 [file pharmaceutics-16-00379-s001.zip › pharmaceutics-2901665-supplementary.pdf]

pharmaceutics

Supplementary Information

Table S1. Optimisation of casting thickness for achieving the target drug loading.

Formulation	Wet casting thickness (μm)	*Dry thickness (μm)	^Dry weight (mg/cm²)	Drug content (μg/cm²)
Control	500	266.7 ± 15.1	12.4 ± 0.4	424.8 ± 24.2
	750	358.3 ± 7.5	19.3 ± 1.0	766.2 ± 31.3
	1000	496.7 ± 17.5	29.0 ± 0.7	1192.7 ± 34.9

*Overall thickness of the patches including the drug-in-adhesive layer, the release liner (75 μm) and the backing membrane (76.2 μm). ^dry weight of the patches including the drug-in-adhesive layer and backing membrane. The results are presented as the mean ± standard deviation (n = 6).

Table S2. Questionnaire for the evaluation of drug-free drug-in-adhesive patches involving healthy volunteers.

Participant ID: ________ Date: __________

1. The patches offered an easy removal from the release liner.

	Extremely disagree	Somewhat disagree	Neither agree nor disagree	Somewhat agree	Extremely agree
Patch-TRAN					
Patch-OA					

2. The patches offered enough initial adhesion so that the patches did not fall off immediately after application.

	Extremely disagree	Somewhat disagree	Neither agree nor disagree	Somewhat agree	Extremely agree
Patch-TRAN					
Patch-OA					

3. The patches offer enough ongoing adhesion for 72 hours. If any of the patches fell off before 72 hours, please indicate the time.

	Extremely disagree	Somewhat disagree	Neither agree nor disagree	Somewhat agree	Extremely agree	Time taken if fell off within 72 hours
Patch-TRAN						
Patch-OA						

4. The patches offered good wear comfort over 72 hours.

	Extremely disagree	Somewhat disagree	Neither agree nor disagree	Somewhat agree	Extremely agree
Patch-TRAN					

**Patch-OA**

5. The patches offered an easy removal from the skin after 72 hours.

	Extremely disagree	Somewhat disagree	Neither agree nor disagree	Somewhat agree	Extremely agree
**Patch-TRAN**					
**Patch-OA**					

6. Indicate any signs or symptoms of local irritations. Please tick all that apply.

		Redness	Itching	Swelling	Others (specify)	No reactions
**Patch-TRAN**	Mild					
	Moderate					
	Severe					
**Patch-OA**	Mild					
	Moderate					
	Severe					

Please write any additional comments below:

____
____
____
____
____

Table S3. The full participants' responses from the questionnaire.

Partici-pant	Q1		Q2		Q3		Q4		Q5		Q6		Comments	
	OA	TR	OA	TR	OA	TR	OA	TR	OA	TR	OA	TR	OA	TR
1	5	5	5	5	5	5	5	5	5	5	Y	N	Mild redness and swelling; subsided after 24 hours; Felt Patch-OA had slightly stronger ad-hesion	
2	5	5	5	5	5	5	5	5	5	5	N	N		
3	5	5	4	4	5	5	5	5	5	5	N	N		
4	5	5	5	5	5	5	5	5	4	4	Y	N	Slight blemish around the applica-tion area	
5	5	5	5	5	5	5	5	5	4	4	N	N	One patch fell off one arm from scratching	
6	5	5	5	5	5	5	5	5	5	5	N	N	Liked the transparency of the patch from the cosmetic perspective	
7	5	5	5	5	4	5	5	5	3	4	N	N	One patch fell off one arm after 48 hours	
8	5	5	5	5	5	4	5	5	5	5	N	N		
9	5	5	5	5	5	5	5	5	3	3	N	N		A bit difficult to re-move the patches after 72 hours
10	5	5	5	5	4	5	5	5	5	5	N	N		
11	4	4	4	4	5	5	5	5	5	5	N	N	Release liner removal could improve; one patch fell off one arm at 60 hours	

OA: Patch-OA; TR: Patch-TRAN; Y: Yes, side effects reported, see the comments section; and N: Nil.
